# Supplementary material for: Capicua regulates neural stem cell proliferation and lineage specification through control of Ets factors
Source: Nat Commun. 2019 May 1;10:2000. doi: 10.1038/s41467-019-09949-6 (PMC6494820; doi:10.1038/s41467-019-09949-6)
Supplement: Supplementary file 1 — Supplementary Information [file 41467_2019_9949_MOESM1_ESM.pdf]

SUPPLEMENTARY INFORMATION

Capicua regulates neural stem cell proliferation and lineage specification through control of Ets factors

Ahmad et al.

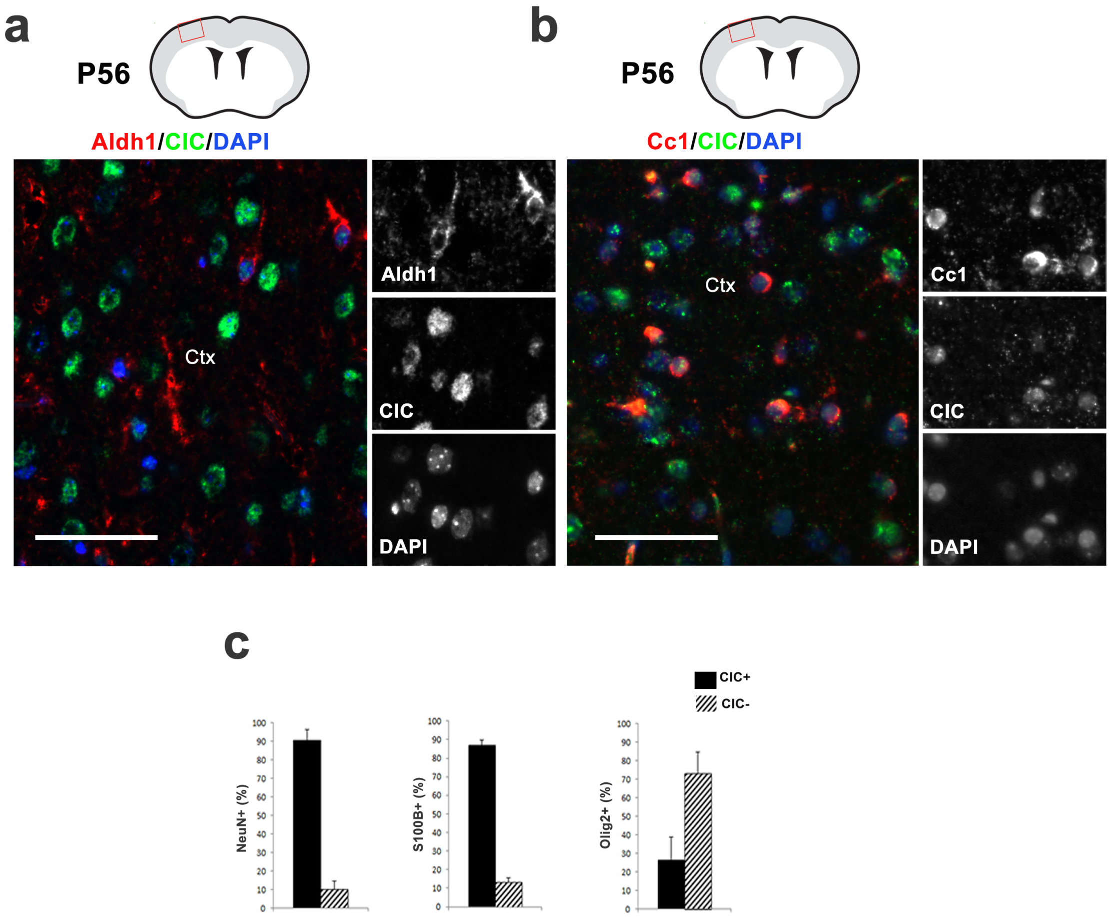

Supplementary Figure 1: Cic expression pattern in mammalian neural lineages. a Cic expression in astrocytes labeled by Aldh1 antibody in P56 cortex. **b** Cic expression in mature oligodendrocytes labeled by Cc1 antibody in P56 cortex. Scale bars: 50µm. **c** Percentages of NeuN+ (neurons), S100B+ (astrocytes) and Olig2+ (oligodendrocytes) having CIC present or absent, when stained with anti-Cic from Thermo and quantitated as a binary variable using arbitrary threshold (data from n=3 animals). Bars indicate mean±SD. Ctx-Cortex.

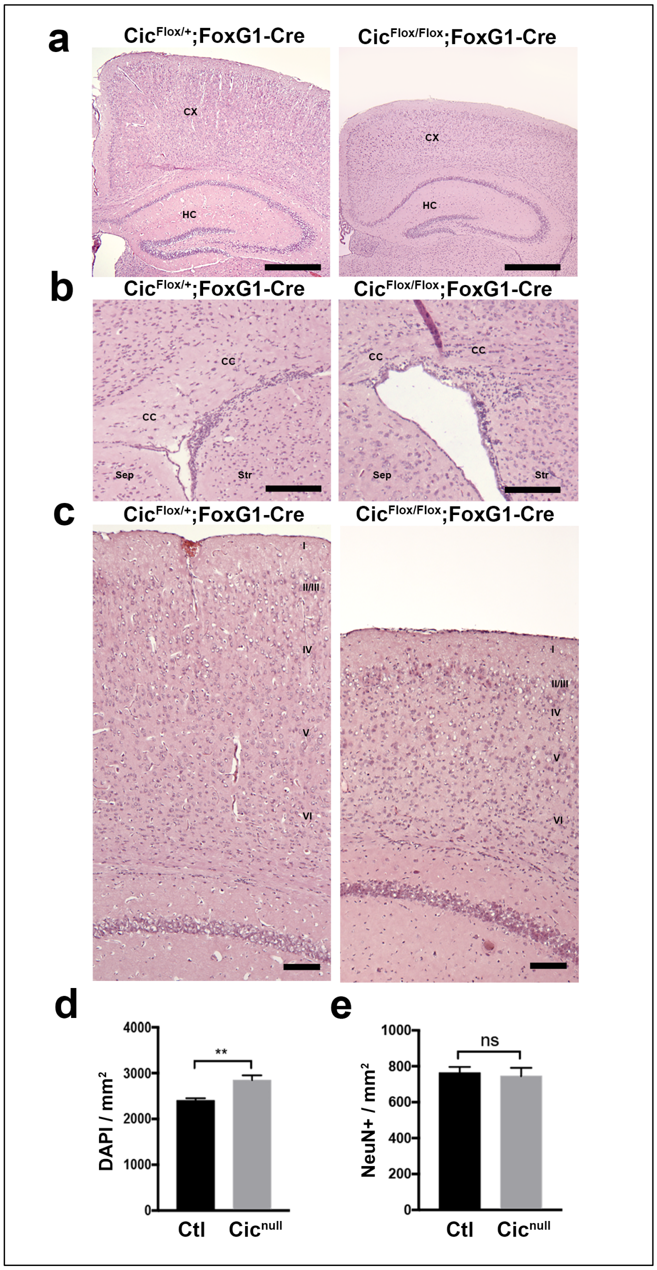

Supplementary Figure 2: Additional phenotype of mice with forebrain *Cic* deletion.
a-c Representative images of H&E stained sections of selected brain regions of mice with CIC heterozygous deletion ($Cic^{Flox/+}$; FoxG1-Cre) or homozygous deletion ($Cic^{Flox/Flox}$; FoxG1-Cre) at P21. **a** Coronal sections through hippocampus and posterior cerebral cortex at level of the anterior nucleus of the thalamus. Homozygous deletion of CIC results in global decreases in volume of cortex, CX, and hippocampus, HC; scale bars 500 µm. **b** Coronal sections through corpus callosum, CC at the level of the anterior commissure. The corpus callosum is thinned with homozygous CIC loss. The subventricular zone, corpus callosum, and adjacent structures show increased cellularity. Scale bar 100 µm. **c** posterior cortex adjacent to hippocampus showing cortical layering. Scale bar 100 µm. CC, corpus callosum; Sep, septal nuclei; Str, striatum. (c) Coronal sections through cortex superior to hippocampus at level of the anterior nucleus of the thalamus show decreased cortical thickness. Scale bar; 100 µm; Cortical layers indicated by I-VI. **d** Cortical cellularity, as measured by DAPI nuclei per mm^2. Data from n = 3 mice per group. **e** Corical neuronal density, as measured by NeuN+ cells per mm^2. Data from n = 3 mice per group. Statistical analyses performed by unpaired t-test. ** p<0.01, ns - not significant.

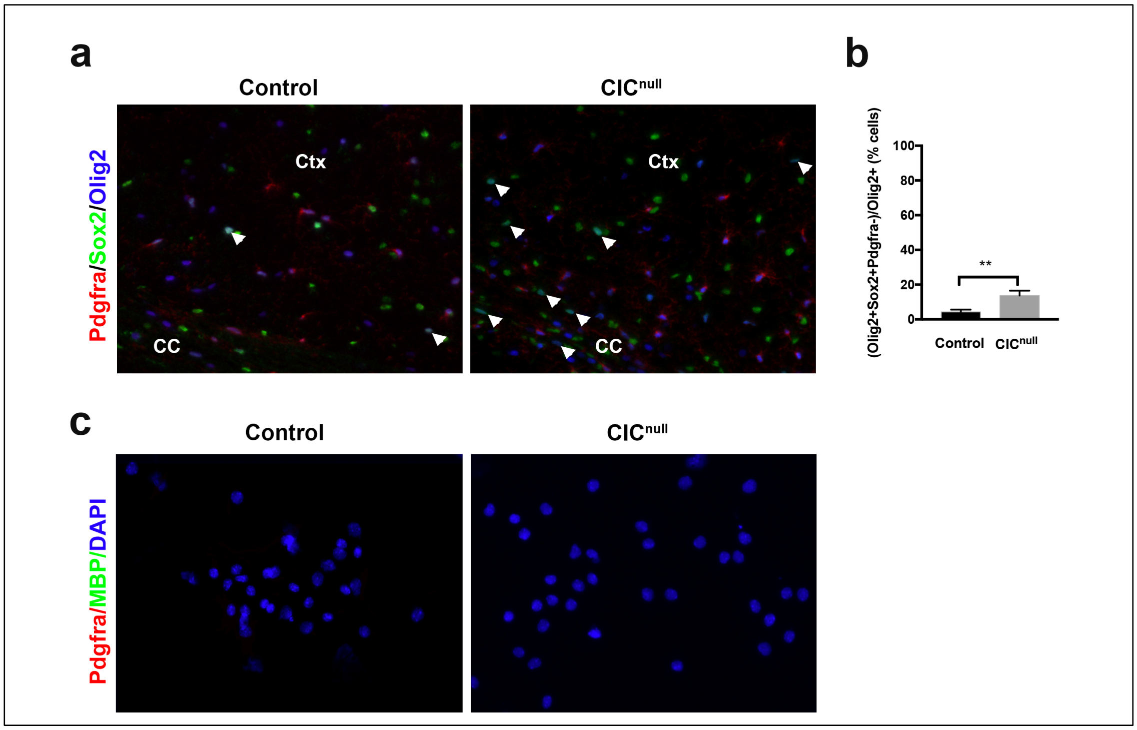

Supplementary Figure 3: a Representative images showing Sox2^{+}Pdgfra^{-}Olig2^{+} cell percentages in control and CIC null mice brains. White arrowheads indicate cells that are Sox2^{+}Olig2^{+}Pdgfra^{-}. **b** Quantitation of Sox2^{+}Pdgfra^{-}Olig2^{+} cell percentages in control and CIC null brains. (CIC$^{Fl/Fl}$;FoxG1$^{Cre/+}$ 13.67±2.89% vs. Control 4.31±1.33%; n=3 mice per group). **c** Representative images showing absence of OPC marker Pdgfra and mature myelinating oligodendrocyte marker Mbp in both control and Cic-null cells under stem cell culture conditions. **p<0.01. CC, corpus callosum, Ctx- cortex. Statistical analyses performed by unpaired t-test.

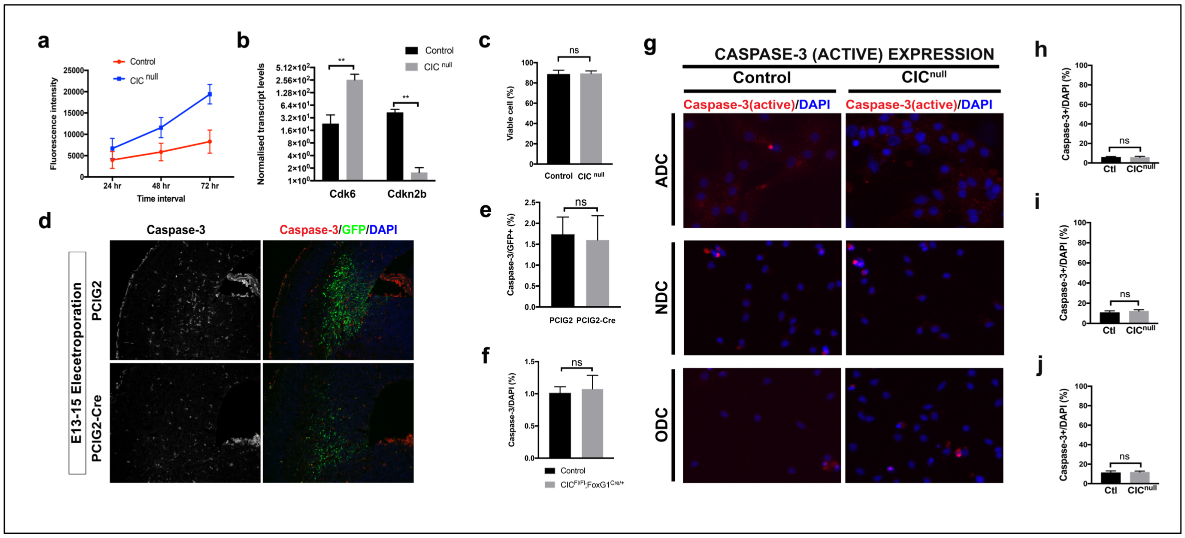

Supplementary Figure 4: CIC loss affects proliferation but not apoptosis.
a Alamar blue assay showing Cic loss induces higher cell proliferation rates at 24 hrs, 48 hrs and 72 hrs after seeding NSCs at equivalent density (n = 3 biological replicates). **b** nanoString analysis of Cic null cells and control cell mRNA showing upregulation of *Cdk6* transcripts and downregulation of *P15INK* transcripts by Cic loss (n = 3 biological replicates). **c** Trypan blue assay shows no difference in the viability of cultured Cic null and control cells when grown in neural stem cell media. **d,e** Cleaved Caspase-3 immunostaining shows no difference in the apoptotic cell numbers within GFP+ cells between Cic null and control cells (n = 5 mice per group). **f** Cleaved Caspase-3 immunostaining shows no difference in the apoptotic cell numbers within GFP+ cells between $Cic^{Fl/Fl};Foxg1^{Cre/+}$ and control brains (n = 5 mice per group). **g** Representative images of caspase-3 immunostaining of all the three neural lineage directed differentiated cells **h-j** Quantifications showing no significant difference in the apoptosis between control and CIC^{null} cell line (n = 3 biological replicates). ADC-Astrocytic differentiation condition, NDC-Neuronal differentiation condition, ODC-Oligodendroglial differentiation condition. Bars indicate mean±SD. ns – not significant, ** p<0.01. All statistical analyses were performed by unpaired t-test.

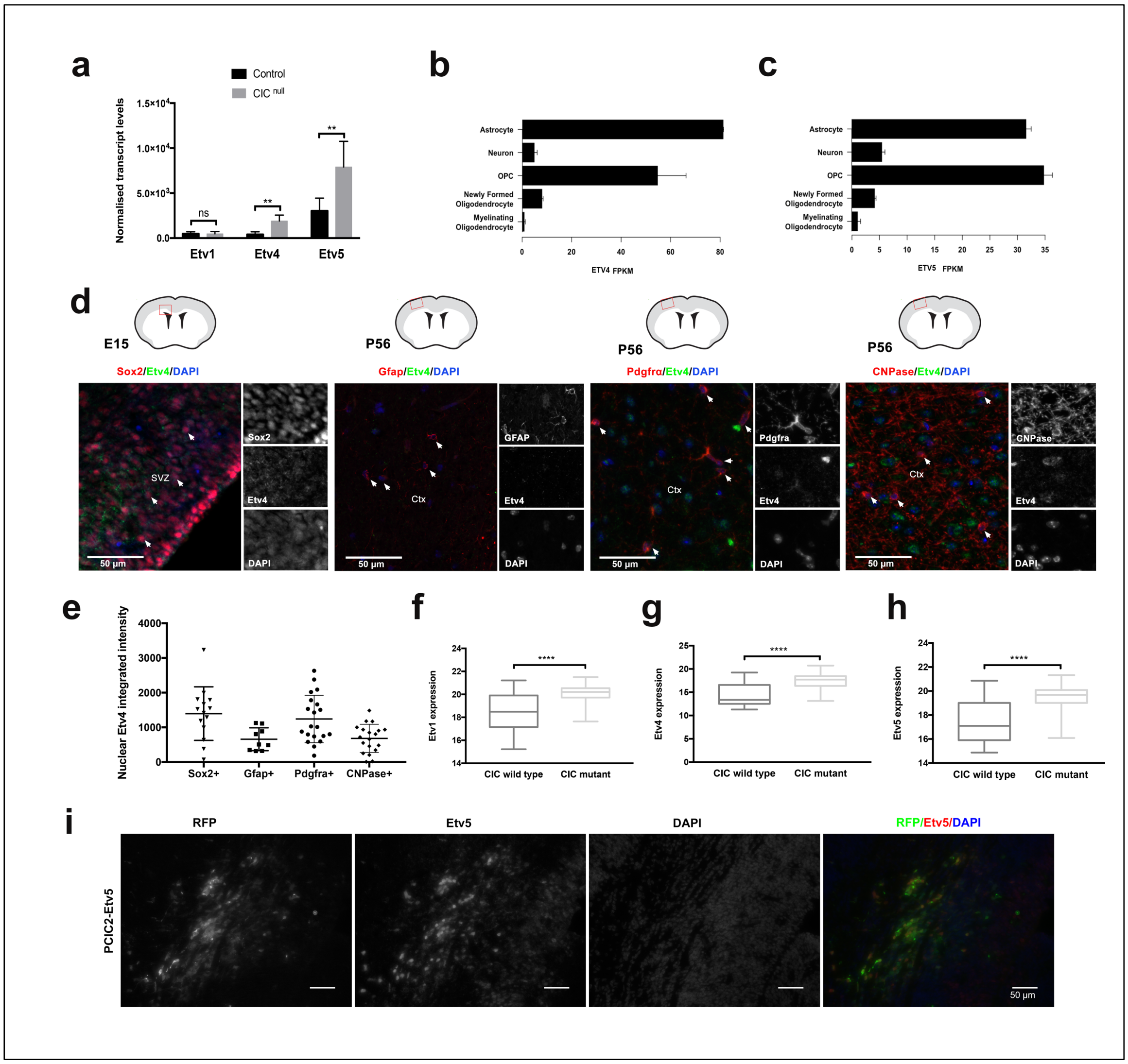

Supplementary Figure 5: Inverse relationship of Ets factors with Cic levels.
a Transcript levels of *Etv1*, *Etv4* and *Etv5* after 10 days of differentiation of mNSCs. **b** Our data is consistent with Barres lab RNAseq data showing high expression of *Etv4* and *Etv5* transcripts in OPCs and astrocytes (Suppementaryl Reference 1). **c** Etv4 protein expression in mouse brain cortical sections across different neural lineages using specific neural lineage markers. **d, e** Representative images and quantification of the Etv4 protein expression across neural lineages. Scale bar: 50µm.
f,g,h Oligodendroglioma patient cohort (TCGA Low Grade Glioma data set) *ETV1*, *ETV4* and *ETV5* transcripts levels between *CIC*-mutant and -wildtype patients indicates higher expression of *ETS* factors in *CIC*-mutant low grade gliomas compared to *CIC*-wildtype low grade gliomas when controlled for *IDH* mutation status. Statistical analyses were done by Student's t test (two tailed). **i** Immunostaining for Etv5 protein confirms that *in-utero* electroporation of *Etv5* construct into mouse forebrain results in localized increased expression of Etv5. Scale bar: 50µm.
SVZ – Sub-ventricular zone, Ctx- Cortex. ns – not significant, ** p<0.01, **** p<0.0001. All the statistical analysis was performed by unpaired t-test.

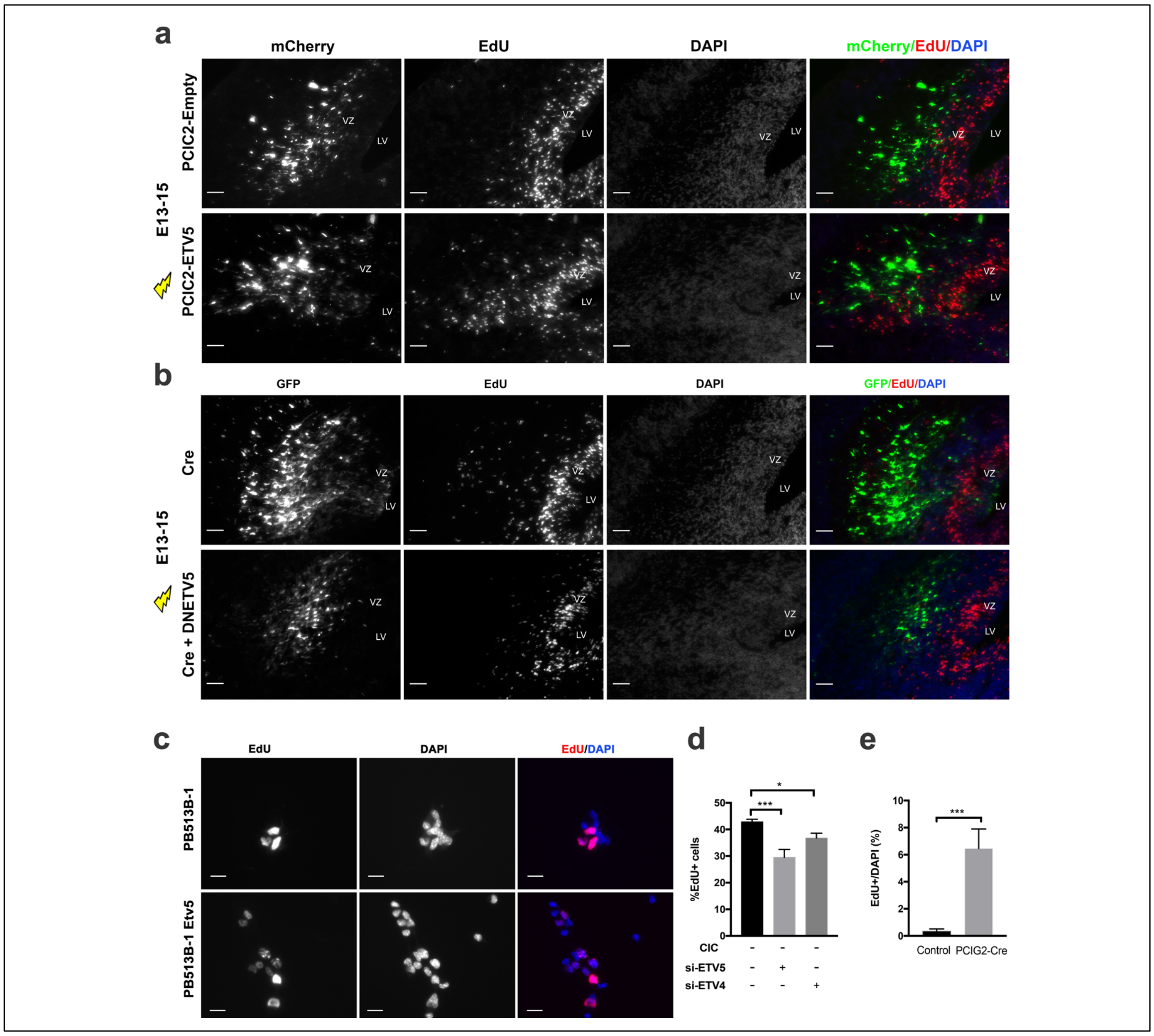

Supplementary Figure 6: The proliferative phenotype induced by CIC loss is Etv5-dependent.

a Representative images showing Etv5 overexpression increases the percentage of cycling EdU+ cells in electroporated mice with Cre electroporation at E13 and analysis at E15. Scale bar: 50µm. **b** Etv5 blockade rescues the high proliferation induced by CIC loss. Scale bar: 50µm.
c Etv5 overexpression increases percentage of EdU+ cells in cultured mNSCs. Scale bar: 10µm
d Quantitation of percentage of EdU+ cells with siRNA knockdown of Etv4 and Etv5 (n = 3 biological replicates per group). Statistical analysis was performed by one-way ANOVA following Tukey's multiple comparisons test. **e** Quantitation of non-cell autonomous proliferative effects of CIC loss after in utero electroporation of Cre from E13 to E15 (n=3). Statistical analyses performed by unpaired t-test. VZ – Ventricular zone, LV – Lateral ventricle. * $p<0.05$, ***$p<0.001$.

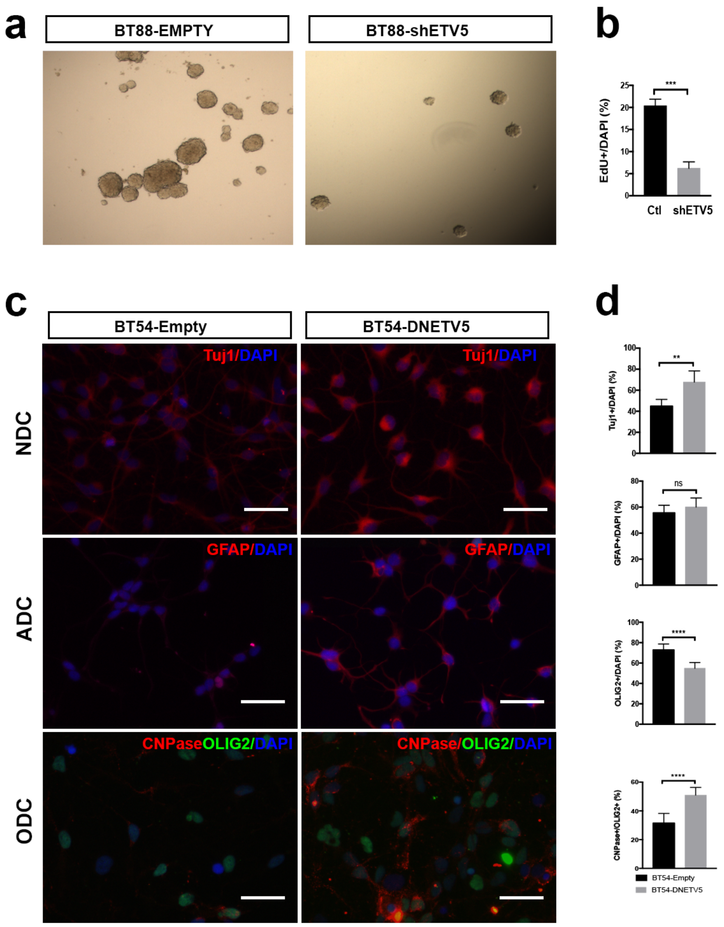

Supplementary Figure 7: Etv5 inhibition in the ODG cell line BT-54 increases responsiveness to differentiation cues.

a Lentiviral knockdown of ETV5 by shRNA showed decrease in sphere numbers and sphere size of BT88 Oligodendroglioma line compared to scramble shRNA . **b** Quantitation of percentage of EdU+ cells with sh-ETV5 versus sh-Scramble. **c** Representative images showing the immunolabelling of BT54-DNETV5 and BT54 control cell lines with neural lineage markers. Scale bar: 50µm. **d** Quantification of the immunostained cells using lineage marker expression of Tuj1 (neurons), Gfap (astrocytes), Olig2 (pan-oligodendroglial), and CNPase (mature oligodendroglial). BT54-DNETV5 cells differentiate into neurons with higher efficiency than control BT54 cells under the same differentiation conditions. There was no significant difference in astrocyte numbers under astrocytic differentiation condition between the BT54-DNETV5 and BT54 control line. BT54-DNETV5 cells undergo terminal oligodendrocyte differentiation and become mature oligodendrocytes with higher efficiency than BT54 controls under oligodendrocytic differentiating condition (n = 3 biological replicates per group). ns – not significant, ** p<0.01, **** p<0.0001. Statistical analyses performed by unpaired t-test.

SUPPLEMENTARY METHODS

The Cancer Genome Atlas data analysis

The Cancer Genome Atlas (TCGA) Lower Grade Glioma (LGG) dataset was downloaded from the online UCSC Xena browser (https://xenabrowser.net/). Tumors that were histologically diagnosed as ODG (grade II or III) or oligoastrocytoma (OA) (grade II or III) that had IDH1 or IDH2 mutations and evidence of 1p19q codeletion (copy number log2(tumor/normal) ≤ -0.5) were included in our analyses. Etv1/4/5 gene expression levels were compared between ODG with wildtype *vs.* mutant CIC.

Immunofluorescence quantitation of neural lineages for presence or absence of CIC expression

A fixed number of Neurons (NeuN+), Astrocytes (GFAP+) and Oligodendrocytes (Olig2+) cells in mice brain sections were counted for the presence or absence of CIC expression. The percentages of CIC+ and CIC- cells in each lineage was ploted on a bar graph.

Alamar Viability assay

10% Alamar Blue reagent (Life Technologies, DAL1100) was added to cells in 96-well plates and allowed to incubate at 37ºC for 6 hours. The fluorescence reading from each well was then assessed ($\lambda_{ex/em}$: 544 nm/590 nm) using a SpectraMax M2^{e} microplate reader (Molecular Devices).

Quantitation of EdU incorporation, non-cell autonomous effects

Equivalent areas of the GFP patch in the IZ (away from VZ/SVZ region) was marked out both in empty construct as well as in Cre electroporated mice brain sections. This area was chosen in order to be able to evaluate similar zones with enough cell numbers in both control electroporation and Cre electroporation conditions. Among GFP- cells in the demarcated zones, EDU+ cells were counted and expressed as a percentage of DAPI stained cells.

Antibodies

Primary antibodies directed against the following were used: CIC (Rabbit Polyclonal, 1:500 IF, 1:1000 WB; A301-204, Bethyl), CIC (Rabbit Polyclonal, 1:100 IF; PA1-46018; Thermo), Olig2 (Mouse Monoclonal, 1:250 IF, 1:1000 WB, MABN50, clone 211F1, Millipore), Olig2 (Rabbit Polyclonal, 1:500, AB9610, Millipore), ETV1 (Mouse Monoclonal, 1:1000 WB, SAB1403794, clone 4C12, Sigma), ETV4 (Rabbit Polyclonal, 1:2000 WB, LS-C98380, LS BioSciences), ETV4 (Rabbit Polyclonal, 1:500 IF 1:2000 WB, ARP32263_P050, AVIVA Systems Biology), ETV5 (Rabbit Polyclonal, 1:1000 WB; sc-22807, Santa Cruz), ETV5 (Polyclonal Rabbit, 1:500 IF, 1:2000 WB, 13011-1-AP, ProteinTech), GFP (Rabbit Polyclonal, 1:500 IF; A11122, Thermo), GFP (Mouse Monoclonal, 1:250 AF, ab38689, clone 6AT316, Abcam), SOX2 (Rabbit Monoclonal, 1:500 IF, ab92494, clone EPR3131, Abcam), Turbo-GFP (Mouse Monoclonal, 1:500 IF, TA150041, clone OTI2H8, Origene), SOX2 (Rabbit Monoclonal, 1:500 IF, 1:1000 WB, #3728, clone C70B1, Cell Signalling), SOX9 (1:500 IF, ab76997, clone 3C10, Abcam), SOX9 (Rabbit Polyclonal, 1:250 IF,1:1000 WB, AB5535, Millipore), PDGFRA (Rabbit Monoclonal, 1:500 IF, 1:1000 WB, 3174S(D1E1E), Cell Signalling), PDGFRA (Goat, Polyclonal, 1:250 IF, AF1062, R&D), CC1 (1:500 IF, APC (Ab-7) (OP80, clone CC1, Millipore), MBP (1:500 IF, ab40390, Abcam), Nestin (1:500 IF, 1:1000 WB, MAB353, Millipore), Tbr1 (1:500 IF, AB2261, Millipore), Tbr2 (Mouse Monoclonal, 1:500 IF, AB15894, Millipore), Neuron-specific beta-III Tubulin (Mouse Monoclonal, 1:500 IF, 1:1000 WB, MAB1195, clone Tuj1, R&D), GFAP (Mouse Monoclonal, 1:800 IF, 1:1000

WB, MAB360, clone GA5, Millipore), GFAP (Rat Monoclonal, 1:500 IF, 345860, clone 2.2B10, Millipore), ALDH1 (Rabbit Polyclonal, 1:250 IF, ab87117, Abcam), NeuN (Mouse Monoclonal, 1:500 IF, MAB377, clone A60, Millipore), Cre (Rabbit Polyclonal, 1:500 IF, 1:2000 WB, NB100-5613, Novus), Ki67 (Rabbit Polyclonal, 1:200; ab15580, Abcam), EdU (61135-33-9, Carbosynth US LLC). For routine immunofluorescence, secondary antibodies were: Alexa Fluor-488, -594 and -633) conjugated species-specific antibodies (Thermo) were used at 1:500 dilution. For the OPAL method (OPAL 4-color kit, Perkin Elmer), secondary antibodies used were anti-mouse HRP polymer (DAKO) and anti-rabbit HRP polymer (DAKO).

ETV5 RNAi

Plasmid pLKO.5 shETV5-A [2] was a gift from William Hahn (Addgene plasmid # 74977 ; http://n2t.net/addgene:74977 ; RRID:Addgene_74977), and was used along with packaging virus to prepare lentiviral particles. The ETV5 shRNA sequence is: CACCTCCAACCAAGATCAAAC. Scramble lentiviral shRNA plasmid [3] and associated ready-to-use lentiviral particles were gifts from David Sabatini (Addgene plasmid # 1864, Addgene viral prep # 1864-LV; http://n2t.net/addgene:1864 ; RRID:Addgene_1864). The sequence of scramble shRNA hairpin is: CCT AAG GTT AAG TCG CCC TCG CTC GAG CGA GGG CGA CTT AAC CTT AGG. Transfected cells were selected with puromycin.

SUPPLEMENTARY REFERENCES

1. Zhang Y, et al. An RNA-Seq transcriptome and splicing database of glia, neurons, and vascular cells of the cerebral cortex. *The Journal of neuroscience : the official journal of the Society for Neuroscience* **34**, 11929-11947 (2014).

2. Wang B, et al. ATXN1L, CIC, and ETS Transcription Factors Modulate Sensitivity to MAPK Pathway Inhibition. *Cell Reports* **18**, 1543-1557 (2017).

3. Sarbassov DD, Guertin DA, Ali SM, Sabatini DM. Phosphorylation and regulation of Akt/PKB by the rictor-mTOR complex. Science **307**, 1098-101 (2015).
