# Supplementary material for: Capicua regulates neural stem cell proliferation and lineage specification through control of Ets factors
Source: Nat Commun. 2019 May 1;10:2000. doi: 10.1038/s41467-019-09949-6 (PMC6494820; doi:10.1038/s41467-019-09949-6)
Supplement: Supplementary file 3 — Reporting Summary [file 41467_2019_9949_MOESM3_ESM.pdf]

Corresponding author(s): Jennifer Chan

Last updated by author(s): Mar 10, 2019

Reporting Summary

Nature Research wishes to improve the reproducibility of the work that we publish. This form provides structure for consistency and transparency in reporting. For further information on Nature Research policies, see Authors & Referees and the Editorial Policy Checklist.

Statistics

For all statistical analyses, confirm that the following items are present in the figure legend, table legend, main text, or Methods section.

n/a	Confirmed	
☐	☒	The exact sample size (n) for each experimental group/condition, given as a discrete number and unit of measurement
☐	☒	A statement on whether measurements were taken from distinct samples or whether the same sample was measured repeatedly
☐	☒	The statistical test(s) used AND whether they are one- or two-sided<br>*Only common tests should be described solely by name; describe more complex techniques in the Methods section.*
☒	☐	A description of all covariates tested
☐	☒	A description of any assumptions or corrections, such as tests of normality and adjustment for multiple comparisons
☐	☒	A full description of the statistical parameters including central tendency (e.g. means) or other basic estimates (e.g. regression coefficient) AND variation (e.g. standard deviation) or associated estimates of uncertainty (e.g. confidence intervals)
☒	☐	For null hypothesis testing, the test statistic (e.g. F, t, r) with confidence intervals, effect sizes, degrees of freedom and P value noted<br>*Give P values as exact values whenever suitable.*
☒	☐	For Bayesian analysis, information on the choice of priors and Markov chain Monte Carlo settings
☒	☐	For hierarchical and complex designs, identification of the appropriate level for tests and full reporting of outcomes
☒	☐	Estimates of effect sizes (e.g. Cohen's d, Pearson's r), indicating how they were calculated

Our web collection on statistics for biologists contains articles on many of the points above.

Software and code

Policy information about availability of computer code

Data collection	GraphPad Prism 8 was used for statistical analyses. Adobe Photoshop Creative Cloud 19.0 release was used for image preparation / colorization. The FIJI open source imaging package (based on Image J) was used for image analysis / quantitation.
Data analysis	Images were processed using Photoshop for orientation, false colorization, and overlay/colocalization. Enumeration of cells positive for cytoskeletal, cytoplasmic or membrane proteins was performed manually by counting positive cells using the Photoshop counting tool. Quantitation of CIC nuclear staining intensity and MBP staining density was performed using FIJI software as follows. Images were first processed using Gaussian blur, then background subtracted. Default threshold limits were used in the threshold tool. Images were converted to binary and watershed was run to separate clumped cells. Nuclear staining was quantitated by using the analysing particles option with separate cut-offs set for each antibody used. For quantitation of MBP expression on tissue sections, FIJI was used by drawing regions of interest on the lateral corpus callosum (cingulum), and the mean integrated density in the regions of interest was calculated.

For manuscripts utilizing custom algorithms or software that are central to the research but not yet described in published literature, software must be made available to editors/reviewers. We strongly encourage code deposition in a community repository (e.g. GitHub). See the Nature Research guidelines for submitting code & software for further information.

Data

Policy information about availability of data

All manuscripts must include a data availability statement. This statement should provide the following information, where applicable:

- Accession codes, unique identifiers, or web links for publicly available datasets
- A list of figures that have associated raw data
- A description of any restrictions on data availability

The authors declare that all data supporting the findings of this study are available within the article and its Supplementary Information files. All biological materials used in this study are available from the corresponding author upon reasonable request.

Field-specific reporting

Please select the one below that is the best fit for your research. If you are not sure, read the appropriate sections before making your selection.

☒ Life sciences ☐ Behavioural & social sciences ☐ Ecological, evolutionary & environmental sciences

For a reference copy of the document with all sections, see nature.com/documents/nr-reporting-summary-flat.pdf

Life sciences study design

All studies must disclose on these points even when the disclosure is negative.

Sample size	No sample size calculation was performed. A minimum of 3 biologically distinct samples were used in our in vitro and in vivo experiments, but the actual number used varies from 3 to 6. For animal studies, many (including survival analysis) are the result of analyses from 5 biologically distinct samples. All n numbers are denoted in the description of the findings.
Data exclusions	No data were excluded from the analyses.
Replication	Experiments throughout the study were performed with independent biology replicates. These were a minimum of 3 biologic replicates per experiment (ranging from 3-6 biologic replicates).
Randomization	There was no randomization in the study.
Blinding	Investigators were not blinded to the identity of the samples during the analyses. To minimize bias, however, counts and quantitations of staining were performed in an automated fashion using FIJI/Image J when possible.

Reporting for specific materials, systems and methods

We require information from authors about some types of materials, experimental systems and methods used in many studies. Here, indicate whether each material, system or method listed is relevant to your study. If you are not sure if a list item applies to your research, read the appropriate section before selecting a response.

Materials & experimental systems

n/a	Involved in the study	
☐	☒	Antibodies
☐	☒	Eukaryotic cell lines
☒	☐	Palaeontology
☐	☒	Animals and other organisms
☐	☒	Human research participants
☒	☐	Clinical data

Methods

n/a	Involved in the study	
☒	☐	ChIP-seq
☒	☐	Flow cytometry
☒	☐	MRI-based neuroimaging

Antibodies

Antibodies used	CIC (Rabbit Polyclonal, 1:500 IF, 1:1000 WB; A301-204, Bethyl), CIC (Rabbit Polyclonal, 1:100 IF; PA1-46018; Thermo), Olig2 (Mouse Monoclonal, 1:250 IF, 1:1000 WB, MABN50, clone 211F1, Millipore), Olig2 (Rabbit Polyclonal, 1:500, AB9610, Millipore), ETV1 (Mouse Monoclonal, 1:1000 WB, SAB1403794, clone 4C12, Sigma), ETV4 (Rabbit Polyclonal, 1:2000 WB, LS-C98380, LS BioSciences), ETV4 (Rabbit Polyclonal, 1:500 IF 1:2000 WB, ARP32263_P050, AVIVA Systems Biology), ETV5 (Rabbit Polyclonal, 1:1000 WB; sc-22807, Santa Cruz), ETV5 (Polyclonal Rabbit, 1:500 IF, 1:2000 WB, 13011-1-AP, ProteinTech), GFP (Rabbit Polyclonal, 1:500 IF; A11122, Thermo), GFP (Mouse Monoclonal, 1:250 AF, ab38689, clone 6AT316, Abcam), SOX2 (Rabbit Monoclonal, 1:500 IF, ab92494, clone EPR3131, Abcam), Turbo-GFP (Mouse Monoclonal, 1:500 IF, TA150041, clone OTI2H8, Origene), SOX2 (Rabbit Monoclonal, 1:500 IF, 1:1000 WB, #3728, clone C70B1, Cell Signalling), SOX9 (1:500 IF, ab76997, clone 3C10, Abcam), SOX9 (Rabbit Polyclonal, 1:250 IF,1:1000 WB, AB5535, Millipore), PDGFRA (Rabbit Monoclonal, 1:500 IF, 1:1000 WB, 3174S(D1E1E), Cell Signalling), PDGFRA (Goat, Polyclonal, 1:250 IF, AF1062, R&D), CC1 (1:500 IF, APC (Ab-7) (OP80, clone CC1, Millipore), MBP (1:500 IF, ab40390, Abcam), Nestin (1:500 IF, 1:1000 WB, MAB353, Millipore), Tbr1 (1:500 IF, AB2261, Millipore), Tbr2 (Mouse Monoclonal, 1:500 IF, AB15894, Millipore), Neuron-specific beta-III Tubulin (Mouse Monoclonal, 1:500 IF, 1:1000 WB, MAB1195, clone Tuj1, R&D), GFAP (Mouse Monoclonal, 1:800 IF, 1:1000 WB, MAB360, clone GA5, Millipore), GFAP (Rat Monoclonal, 1:500 IF, 345860, clone 2.2B10, Millipore), ALDH1 (Rabbit Polyclonal, 1:250 IF, ab87117, Abcam), NeuN (Mouse Monoclonal, 1:500 IF, MAB377, clone A60, Millipore), Cre (Rabbit Polyclonal, 1:500 IF, 1:2000 WB, NB100-5613, Novus), Ki67 (Rabbit Polyclonal, 1:200; ab15580, Abcam), EdU (61135-33-9, Carbosynth US LLC). For routine immunofluorescence, secondary antibodies were: Alexa Fluor-488, -594 and -633) conjugated species-specific antibodies (Thermo) were used at 1:500 dilution. For the OPAL method (OPAL 4-color kit, Perkin Elmer), secondary antibodies used were anti-mouse HRP polymer (DAKO) and anti-rabbit HRP polymer (DAKO).

Validation

All antibodies were either validated by the manufacturer or cited in previous publications. For the CIC antibodies in particular, we validated them on our Cic knockout cells cells and tissues versus wildtype / endogenous expression.

Eukaryotic cell lines

Policy information about cell lines

Cell line source(s)

The BT88 and BT54 cell lines are patient derived brain tumour lines that were generated at the University of Calgary by Dr. Sam Weiss and Dr. J. Gregory Cairncross, with our direct collaboration. They have been reported previously in the literature, have been extensively sequenced, and we have cited the references for the lines.

Authentication

BT88 and BT54 are routinely authenticated by STR profiling using the Identifiler Plus Kit (Thermo), with profiles matched to the reference profile of the primary patient material. Authentication is performed at least every 6 months. We have not had any cross-contamination while using the lines.

Mycoplasma contamination

BT88 and BT 54 are tested for mycoplasma once per month. We have not had any mycoplasma contamination of these lines.

Commonly misidentified lines
(See ICLAC register)

Name any commonly misidentified cell lines used in the study and provide a rationale for their use.

Animals and other organisms

Policy information about studies involving animals; ARRIVE guidelines recommended for reporting animal research

Laboratory animals

This study uses CIC conditional knockout mice. They are on a C57Bl6 background. Both males and females were used in the study. Ages of analysis for the CIC mice included embryonic ages from E13 onward, early postnatal, and later ages (P21 and P56). For crossing with the CIC-CKO animals, we used the FoxG1-cre line (available from Jax); both male and female were used. For orthotopic xenograft experiments, NOD-SCID females were used.

Wild animals

The study did not involve wild animals.

Field-collected samples

The study did not involve samples collected from the field.

Ethics oversight

The animal work in this project was approved by the University of Calgary's Animal Care Committee (protocol AC16-0266) in compliance with the Guidelines of the Canadian Council of Animal Care.

Note that full information on the approval of the study protocol must also be provided in the manuscript.

Human research participants

Policy information about studies involving human research participants

Population characteristics

This study uses human-derived materials. The cell lines BT88 and BT54 were patient derived materials from two human oligodendroglioma patients.

Recruitment

The patients from which the cell lines were derived had consented to participation in the Clark Smith Tumour Bank, an ethics-approved brain tumour biobank at the University of Calgary. The consent for bank participation also includes a section on consent for material to be used to generate cell lines. A separate ethics-approved project to generate lines from gliomas is what generated the lines from the de-identified material.

Ethics oversight

Health Research Ethics Board of Alberta

Note that full information on the approval of the study protocol must also be provided in the manuscript.
